# Low‐Dose Oral Minoxidil and Associated Adverse Events: Analyses of the FDA Adverse Event Reporting System (FAERS) With a Focus on Pericardial Effusions

**DOI:** 10.1111/jocd.16574

**Published:** 2024-09-26

**Authors:** Aditya K. Gupta, Mary A. Bamimore, Husam Abdel‐Qadir, Greg Williams, Antonella Tosti, Vincent Piguet, Mesbah Talukder

**Affiliations:** ^1^ Mediprobe Research Inc. London Ontario Canada; ^2^ Division of Dermatology, Department of Medicine, Temerty Faculty of Medicine University of Toronto School of Medicine Toronto Ontario Canada; ^3^ Division of Cardiology Women's College Hospital and University Health Network Toronto Ontario Canada; ^4^ Department of Medicine and Institute of Health Policy, Management and Evaluation University of Toronto Toronto Ontario Canada; ^5^ Farjo Hair Institute London UK; ^6^ Miller School of Medicine University of Miami Miami Florida USA; ^7^ Division of Dermatology Women's College Hospital Toronto Ontario Canada; ^8^ School of Pharmacy BRAC University Dhaka Bangladesh

**Keywords:** adverse effect, alopecia, androgenetic alopecia, minoxidil

## Abstract

**Background:**

Low‐dose oral minoxidil (LDOM) is used to treat hair loss, but the literature on its safety profile is relatively sparse.

**Aims:**

Using the FDA Adverse Event Reporting System (FAERS) database, we determined signals for adverse events (AEs) with LDOM use.

**Methods:**

Four sets of case/noncase study disproportionality analyses were conducted to determine reporting odds ratio (ROR) for 10 AEs including pericardial effusion (PE). The oral minoxidil dose ranges were: (i) ≤1.25 mg (i.e., 0–1.25 mg), (ii) ≤2.5 mg (i.e., 0–2.5 mg), (iii) ≤5 mg (i.e., 0–5 mg), and (iv) ≤10 mg (i.e., 0–10 mg).

**Results:**

For ≤1.25 mg, we detected a signal for PE (ROR = 16.41, 95% CI: 2.29, 117.37, *p* < 0.05). For ≤2.5 mg, the analyses detected a signal for PE (ROR = 13.30, 95% CI: 5.96, 29.68, *p <* 0.05); the ROR in the absence of cardiac impairment was 5.34 (95% CI: 1.33, 21.37, *p <* 0.05); in the presence of cardiac impairment, the ROR was 49.42 (95% CI: 18.27, 133.66, *p <* 0.05). A signal for PE was also detected at ≤5 and ≤10 mg. For PE, there was a significant (*p <* 0.05) association with a patient outcome of “life threatening” only at the ≤10 mg dose range.

**Conclusions:**

Our study, the first FAERS‐based signal detection study for LDOM, found significant associations between LDOM use and several AEs. In the absence of causal evidence, these correlations warrant more attention regarding safe use of LDOM. Until more safety data are available, we recommend using LDOM at the lowest effective dose (≤5 mg/day).

## 1 Introduction

Oral minoxidil was inceptively used to treat hypertension; more recently it has been prescribed off‐label for male pattern hair loss (MPHL) and female pattern hair loss (FPHL) [1, 2]. Doses above 10 mg per day have been used to treat severe refractory hypertension [3], while hair regrowth has been associated with lower doses. Some have indicated that LDOM is a dose of ≤10 mg daily [1]; others have suggested a dose of ≤5 mg; however, there is no uniformly accepted definition of LDOM. The literature suggests that FPHL patients are prescribed relatively lower doses of minoxidil (range: 0.25–2.5 mg/day) compared with MPHL patients (range: 1.25–5.0 mg/day), possibly because females are more prone to LDOM‐associated hypertrichosis and systemic side effects than males [2, 4]. Additionally, females generally have lower body weight than males.

Topical minoxidil was licensed by the FDA for MPHL in 1988 and for FPHL in 1991, well before the popularity of off‐label use [5]. While topical minoxidil (2% and 5%) has been FDA‐approved for over 30 years, the peer‐reviewed literature for LDOM and hair loss is under a decade old. Beach coined the descriptor “*5 C's of oral therapy*” to summarize five advantages of the oral route versus topical application: (1) “*cosmesis*,” (2) “*convenience*,” (3) “*co‐therapy*,” (4) “*cost‐savings*,” and (5) “*compliance*” [6].

With the use of LDOM for alopecia increasing, more data has begun to accumulate regarding adverse events (AEs). In Vañó‐Galván et al.'s retrospective, cohort study (857 males, 1612 females) on the safety of LDOM (0.03–15 mg) seven AEs were observed: dizziness (light headedness), fluid retention, headache, hypertrichosis, insomnia, periorbital edema, and tachycardia [2]; other AEs such as hypotension, pericardial effusion, and peripheral edema have also been documented previously.

Since its inception in 1969, The US Food and Drug Administration Adverse Event Reporting System (FAERS)—which is a database that houses global information pertaining to voluntarily reported AEs—had become a *sine qua non* data source for pharmacovigilance studies. Analyses of FAERS data has not only contributed to the safety literature of many drugs, but is also a desideratum in the postmarketing withdrawal of numerous medicines [7, 8]. The expansion of a therapy's safety profile hinges on observational studies: by design, randomized controlled trials (RCTs) are typically not statistically powered enough for the identification of rare AEs.

To date, the work by Ortega‐Quijano et al. is the only study that investigated the safety of LDOM using the FAERS database [9]. However, their work was solely descriptive (i.e., no disproportionality analysis of any sort). We determined whether FAERS data could detect signals for AEs that case reports had associated with LDOM.

## 2 Methods

The conduct of our work was guided by the “*STrengthening the Reporting of OBservational studies in Epidemiology”* (STROBE) recommendations [10] as well as guidelines of the “*Reporting of A Disproportionality analysis for drUg Safety signal detection using spontaneously reported adverse events in Pharmacovigilance”* (READUS‐PV) [11].

### 2.1 Data Sources

The FAERS database is constituted of seven anonymized datasets that each have a common variable for the identification of case reports. Furthermore, the FAERS database is updated quarterly, hence an observation period of 1 year constitutes data from 4 quarters. Our entire analyses involved five of the seven datasets, namely, the “DEMO,” “DRUG,” “OUTC,” “REAC” and “INDI” files [12]. Demographic information (e.g., age and sex) are contained within the DEMO dataset; drug‐related details (e.g., drug name, active ingredient, and dose) are within the ‘DRUG’ file. The DRUG files corresponding to our observation period (i.e., 2016–2023, inclusive) were structured in such a way that analyzing drug information was straightforward, compared with the structure in former quarters. For instance, “dose amount” and “active ingredient” are stand‐alone variables in the DRUG file that were not present in the former quarters. The ‘REAC’ file has information on adverse events—where each is described according to the preferred term (pt) nomenclature of the Medical Dictionary for Regulatory Activities (MedDRA, version 27.0) [13]. The “OUTC” file details patient outcomes (e.g., death, and hospitalization) and the ‘INDI’ file corresponds to information regarding indications for drug use [12].

We used five of the seven datasets because of none of our analyses required the other two; moreover, errors due to double counting are avoided when there is no merging of unnecessary files.

### 2.2 Analyses Plan

All analyses were conducted using the *R* software (version 4.3.2); we used the *dplyr* [14] and *sqldf* [15] *R* packages for data mining. Alpha (cutoff for significance level) was 5%. Across all analyses, the observation period was for 8 years, i.e., 2016–2023 (inclusive).

We examined 10 specific adverse events that case reports [16] had associated with LDOM, namely, dizziness, fluid retention, headache, hypertrichosis, hypotension, insomnia, pericardial effusion, periorbital edema, peripheral edema, and tachycardia. Given that the MedDRA classification system (version 27.0) distinguishes peripheral edema (pt MedDRA Code = 10030124) from fluid retention (pt MedDRA Code = 10016807), we treated the two as distinct entities for analyses purposes; moreover, the International Classification of Disease (ICD) system of the World Health Organization (WHO) also distinguishes between the two.

For the treatment of PHL, many physicians would consider an oral minoxidil dose of ≤5 mg/day. We conducted four sets of analyses according to the following dose ranges: (i) ≤ 1.25 mg minoxidil (i.e., 0–1.25 mg), (ii) ≤2.5 mg minoxidil (i.e., 0–2.5 mg), (iii) ≤5 mg minoxidil (i.e., 0–5 mg), and (iv) ≤10 mg minoxidil (i.e., 0–10 mg). For each dose range, we performed the following three analyses: (i) conducted disproportionality analyses to estimate reporting odds ratios (and corresponding 95% confidence interval [CI]) for LDOM and each of the 10 AEs (i.e., Analyses 1), (ii) “stratified” disproportionality analyses for pericardial effusion and LDOM according to presence (i.e., indication) and absence (i.e., no indication) of renal and cardiac impairment (Analyses 2), and (iii) disproportionality analyses for LDOM and patient outcomes (i.e., congenital anomaly, death, disability, hospitalization, occurrence of life‐threatening condition(s), and requisition of medical intervention to prevent irreversible damage) across reports of AEs for which a signal was detected (Analyses 3).

Regarding Analyses 2, we used the “Breslow‐Day test” to determine whether point estimates across the different strata were significantly (*p <* 0.05) different; the stratification analyses were performed because renal and cardiac impairment are often observed with pericardial effusion in clinical practice; moreover, the stratification analyses were feasible as the FAERS database provides indication data through the “INDI” files. For Analyses 2, our interpretations for renal and cardiac impairment are in the context of “indication” and not “diagnosis.” For the purpose of analyses, renal and cardiac impairment were, intuitively, “composite” conditions; for example, indication for renal impairment—as per preferred terms of MedDRA—included “renal tubular dysfunction,” “complications of transplanted kidney,” and so forth.

### 2.3 Disproportionality Analysis

For our disproportionality analyses, we chose the ROR for signal detection as it is the metric of choice in numerous pharmacovigilance studies [17]. Computation of a ROR and its corresponding 95% CI starts with obtaining counts of the following: (i) number of reports where intervention of interest resulted in the occurrence of a specific AE, (ii) number of reports where intervention of interest resulted in the occurrence of all other AEs, (iii) number of reports where other interventions resulted in the occurrence of a specific AE, and (iv) number of reports where other interventions resulted in the occurrence of all other AEs. A signal is detected when the values of ROR and the lower bound of its 95% CI are each above 1.00 [18]. For the current study, a signal is actually “signal of disproportionate reporting” which corresponds to a statistically positive correlation between the AE and the drug if interest. Such signals in themselves do not serve as causal evidence.

The ROR and its 95% CI were calculated as per the following equations:

Contingency table for count data used in the computation of a ROR and its 95% CI

(1.1) $$\begin{array}{ccc} & \text{Intervention of interest} & \mathrm{All}\;\text{other drugs}\\ \text{Outcome}\left(s\right)\;\text{of interest} & a & b\\ \mathrm{All}\;\text{other outcome}\left(s\right) & c & d \end{array}$$

(1.2) $$\text{Reporting odds ratio}\;\left(\mathrm{ROR}\right)=\frac{a/c}{b/d}$$

(1.3) $$\begin{array}{c} \text{Lower bound of}\;95\%\text{confidence interval for}\;\mathrm{ROR}\\ =e^{\ln\left(\mathrm{ROR}\right)-1.96\;\sqrt{\frac{1}{a}+\frac{1}{b}+\frac{1}{c}+\frac{1}{d}}} \end{array}$$

(1.4) $$\begin{array}{c} \text{Upper bound of}\;95\%\text{confidence interval for}\;\mathrm{ROR}\\ =e^{\ln\left(\mathrm{ROR}\right)+1.96\;\sqrt{\frac{1}{a}+\frac{1}{b}+\frac{1}{c}+\frac{1}{d}}} \end{array}$$

The literature guided the data mining process for our disproportionality analyses—including the deduplication steps; we produced flow charts that provided an overview of these steps. In our results for Analyses 1 to 3, the ROR corresponding to counts of reports for outcome of interest across drug of interest. Equations ((1.1), (1.2), (1.3), (1.4)) corresponds to a case/noncase disproportionality analysis where case (i.e., a/c) corresponds to number of reports retrieved for drug of interest and noncase (b/d) corresponds to every other drug. For example, in the analyses pertaining to ≤10 mg minoxidil, case, referred to reports pertaining to ≤10 mg, while noncases corresponded to every drug but minoxidil.

## 3 Results

### 3.1 Descriptive Summaries

For the ≤10 mg dose range details of patients' age and sex, as well as reporters' profession—across each AE, —are summarized in Appendix A of the supplement; across reports of most of the AEs, majority of cases were male. Dizziness, fluid retention, and insomnia were mostly reported by consumers—while headache, hypotension, hypertrichosis, pericardial effusion, peripheral edema, and tachycardia were chiefly reported by health professionals (e.g., physicians and pharmacists). Reports of periorbital edema by LDOM use were not identified (Appendix A of the supplement). Across the adverse events, reports of hypertrichosis had the youngest mean age (Appendix A of the supplement). Tables S1–S4 in supplement present frequencies for the occurrence of each AE vs. all other AEs across LDOM use and nonLDOM use, for the four dose ranges.

### 3.2 Analytical Results

In Appendix B of the supplement, we provided presented flow charts.

**FIGURE 1:**
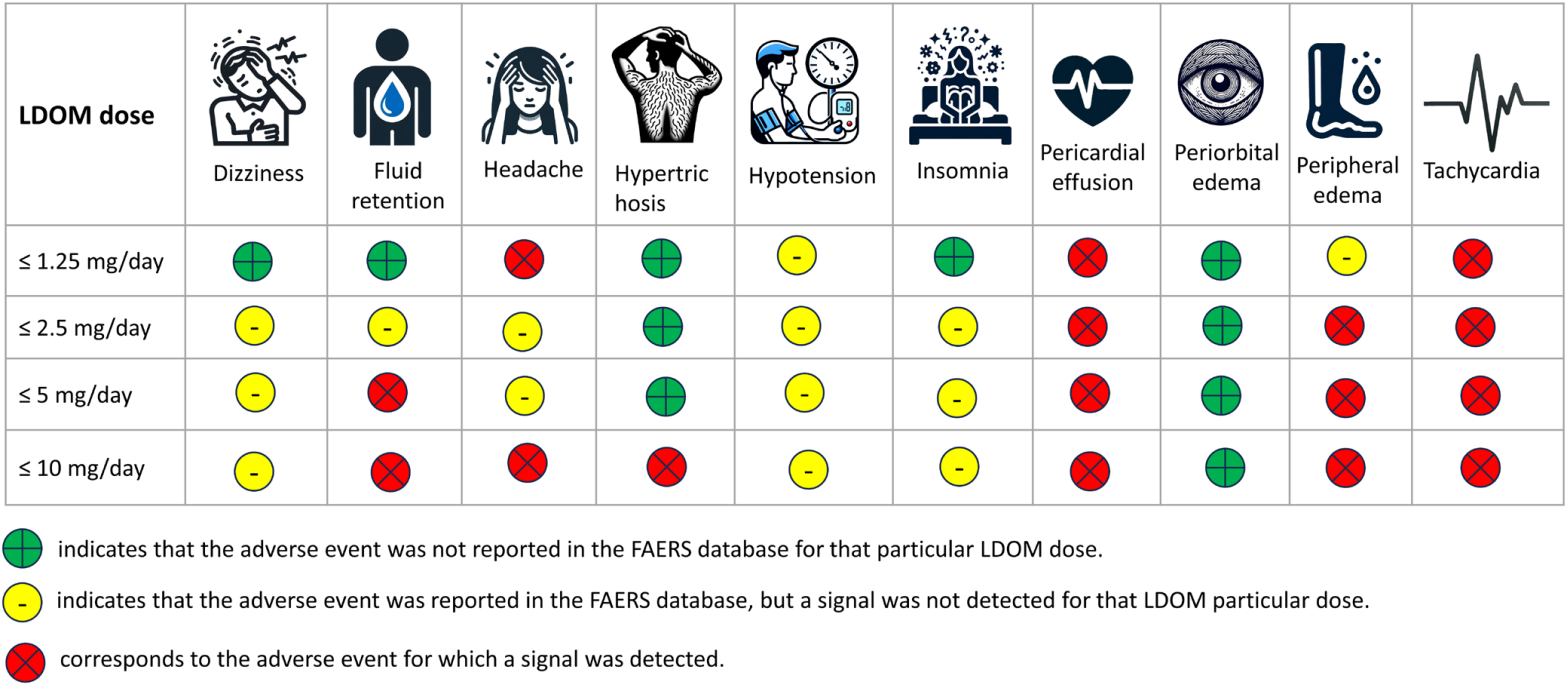
FAERS‐based analytical study identified signals for potential adverse events associated with LDOM (2016–2023).

Our disproportionality analyses detected signals across the various dose ranges; we detected the following: ≤1.25 (3 signals), ≤2.5 (3 signals), ≤5 (4 signals), and ≤10 mg (6 signals). For ≤1.25 mg signal was detected for tachycardia and headache; though a signal was detected for pericardial effusion, it is important to note a convention that is sometimes followed: ROR is sometimes not computed when the number of cases (i.e., which would correspond to “*A*” in equation 1.1) is less than 3; so the signal we found at ≤1.25 mg for pericardial effusion should be interpreted with caution. For ≤2.5 mg and ≤ 5 mg, signals were detected for pericardial effusion, peripheral edema, and tachycardia; signal detection for hypertrichosis occurred with ≤10 mg dose range. Our analyses detected a signal for fluid retention with ≤5 mg and ≤10 mg dose ranges (Table 1). Figure 1 summarizes the LDOM‐associated adverse events for which signals were detected; however, caution should be taken when interpreting and/or using Figure 1 in any decision making because of the “sampling”: arguably, findings from spontaneously reported data like FAERS are less generalizable than a those from a prospective cohort study. Because each analysis constituted data from different sources, the sample size (i.e., number of observations) for each analysis differed. For instance, the stratified analyses (i.e., Analysis 3) used distinct reports across merged “DEMO,” “DRUG,” “REAC,” and “INDI” files; whereas, the disproportionality analyses for LDOM use and AEs (i.e., Analysis 1) used distinct reports across merged “DEMO,” “DRUG,” and “REAC” files.

**TABLE 1:** Association between LDOM and AEs across 2016–2023 (inclusive).

AE	LDOM ≤1.25 mg (*N* = 97 842 386) (*N* ^a^ = 130)				LDOM ≤2.5 mg (*N* = 97 843 217) (*N* ^b^ = 961)				LDOM ≤5 mg (*N* = 97 843 530) (*N* ^c^ = 1274)				LDOM ≤10 mg (*N* = 97 844 330) (*N* ^d^ = 2074)			
	No. of cases^a^	ROR	Lower bound of 95% CI	Upper bound of 95% CI	No. of cases^a^	ROR	Lower bound of 95% CI	Upper bound of 95% CI	No. of cases^a^	ROR	Lower bound of 95% CI	Upper bound of 95% CI	No. of cases^a^	ROR	Lower bound of 95% CI	Upper bound of 95% CI
Dizziness	*—*	*—*	*—*	*—*	10	1.437	0.771	2.679	13	1.409	0.816	2.433	22	1.465	0.963	2.23
Fluid retention	*—*	*—*	*—*	*—*	2	2.006	0.501	8.03	5	3.789	1.575	9.118	7	3.257	1.551	6.839
Headache	9	8.363	4.249	16.46	12	1.422	0.805	2.513	15	1.34	0.806	2.229	27	1.500	1.026	2.193
Hypertrichosis	*—*	*—*	*—*	*—*	*—*	*—*	*—*	*—*	*—*	*—*	*—*	*—*	3	49.747	16.022	154.47
Hypotension	2	3.796	0.939	15.34	4	1.016	0.381	2.711	5	0.958	0.398	2.304	13	1.533	0.889	2.644
Insomnia	*—*	*—*	*—*	*—*	5	1.115	0.463	2.685	5	0.84	0.349	2.021	7	0.722	0.344	1.516
Pericardial effusion	1	16.408	2.294	117.374	6	13.299	5.96	29.678	11	18.435	10.183	33.376	19	19.570	12.456	30.748
Periorbital edema	*—*	*—*	*—*	*—*	*—*	*—*	*—*	*—*	*—*	*—*	*—*	*—*	*—*	*—*	*—*	*—*
Peripheral edema	1	3.705	0.518	26.502	8	4.012	2.001	8.046	12	4.545	2.574	8.024	15	3.482	2.096	5.786
Tachycardia	9	30.837	15.667	60.699	13	5.685	3.289	9.827	15	4.940	2.969	8.219	19	3.834	2.44	6.023

*Note:* No. of cases: Number of cases correspond to number of reports retrieved for the respective AE and the respective dose range of minoxidil. The information presented in this table was produced from analyses of the US Food and Drug Administration Adverse Event Reporting System (FAERS) database. This table shows the association between LDOM and each of the 10 AEs. Gray‐colored cells with bolded text correspond to adverse events for which signals were detected. A signal is detected when the ROR and lower bound of the 95% CI are each greater than 1; in other words, a signal occurs when the 95% CI does not include the null value (i.e., 1.00). For the gray‐colored cells with bolded text, *p <* 0.05. N = sample size (i.e., number of observations (or number of reports) which ROR computation was based on). N^a^ = number of reports pertaining to ≤1.25 mg. N^b^ = number of reports pertaining to ≤2.5 mg. N^c^ = number of reports pertaining to ≤5 mg. N^d^ = number of reports pertaining to ≤10 mg.

Abbreviations: AE, adverse event; CI, confidence interval; LDOM, low‐dose oral minoxidil; mg, milligram; ROR, reporting odds ratio.

^a^ RORs for where number of cases is less than three should be interpreted with caution because in some conventions, ROR is to be calculated when no. of cases is at least three; this convention is not always followed, so that is why we noted this.

For peripheral edema, we found significant (*p <* 0.05) associations between oral minoxidil (≤2.5 mg, ≤5 mg, and ≤10 mg) and requirement of medical intervention (to prevent permanent impairment) across each of the three dose ranges (Table 2). For pericardial effusion, an association between oral minoxidil and life‐threatening outcome(s) was found only for the ≤10 mg dose range (Table 2).

**TABLE 2:** Association between LDOM and patient outcomes, across 2016–2023 (inclusive).

AE	Serious patient outcomes					
	Required intervention^a^, ROR (95% CI)	Life‐threatening, ROR (95% CI)	Hospitalization, ROR (95% CI)	Disability, ROR (95% CI)	Death, ROR (95% CI)	Congenital anomaly, ROR (95% CI)
LDOM ≤ 1.25 mg						
Tachycardia (*n* = 400 972)			0.179		0.884	
	***—***	*—*	(0.023, 1.398)	*—*	(0.114, 6.901)	*—*
LDOM ≤ 2.5 mg						
Pericardial effusion (*n* = 82 677)		1.548	1.373			
	*—*	(0.197, 12.22)	(0.398, 4.743)	*—*	*—*	*—*
Peripheral edema (*n* = 327 125)	**224.248** **(28.248, 1780.245) No. of cases = 1**	***—***	1.175(0.332, 4.164)	*—*	*—*	*—*
				
Tachycardia (*n* = 400 978)	***—***	0.553	0.384			
		(0.074, 4.164)	(0.111, 1.334)	*—*	*—*	*—*
LDOM ≤5 mg						
Fluid retention (*n* = 151 538)			1.536			
	*—*	*—*	(0.31, 7.606)	*—*	*—*	*—*
Pericardial effusion (*n* = 82 684)		1.858	1.221			
	*—*	(0.425, 8.125)	(0.471, 3.164)	*—*	*—*	*—*
Peripheral edema (*n* = 327 129)	**155.249 (20.191, 1193.730) No. of cases = 1**		0.979			
		***—***	(0.329, 2.922)	*—*	*—*	*—*
Tachycardia (*n* = 400 980)		0.491	0.477			
	***—***	(0.066, 3.677)	(0.159, 1.438)	*—*	*—*	*—*
LDOM ≤10 mg						
Fluid retention (n = 151 541)			1.919			
	*—*	*—*	(0.516, 7.146)	*—*	*—*	*—*
Headache (*n* = 1 119 504)		0.967	1.363	1.344		
	*—*	(0.133, 7.05)	(0.674, 2.758)	(0.324, 5.586)	*—*	*—*
Pericardial effusion (*n* = 85 592)		**2.903 (1.107, 7.61) No. of cases = 5**	1.69			
	*—*		(0.813, 3.514)	*—*	*—*	*—*
Peripheral edema (*n* = 327 134)	**112.124 (14.88, 844.88) No. of cases = 1**		1.282			
		*—*	(0.516, 3.187)	*—*	*—*	*—*
Tachycardia (*n* = 400 984)		0.402	0.954			
	*—*	(0.055, 2.979)	(0.405, 2.25)	*—*	*—*	*—*

*Note:* No. of cases: Number of cases correspond to number of reports retrieved for the respective AE and the respective dose range of minoxidil.

Abbreviations: AE, adverse event; CI, confidence interval; low‐dose oral minoxidil; mg, milligram; *n*, number of observations/sample size that ROR calculation was based on; LDOM.

^a^ Required intervention to prevent permanent impairment/damage. The information presented in this table was produced from analyses of the United States Food and Drug Administration Adverse Event Reporting System (FAERS) database. This table summarizes the association between serious patient outcomes (i.e., hospitalization, disability, etc.) and LDOM across the respective AE group for which signals were detected. The darker gray‐colored cells with bolded text correspond to significant association between LDOM and the serious patient outcome across reports of the respect AE. For example, across reports of pericardial effusion and LDOM ≤ 10 mg (*n* = 82 696), use of LDOM up to 10 mg is significantly (*p* < 0.05) associated with life‐threatening outcomes.

We found that oral minoxidil dose ranges (≤10, ≤5, and ≤2.5 mg) were significantly (*p <* 0.05) associated with pericardial effusion in both the presence and absence of indication for cardiac impairment (Table 3); furthermore, the test for homogeneity across the two strata (i.e., the Breslow‐Day test) showed that the association in the presence of indication for cardiac impairment was significantly higher than the association in the absence of indication for cardiac impairment (Table 3). Disproportionality analyses for oral minoxidil and pericardial effusion in the presence of renal impairment were not conducted due to lack of data; however, the analyses for absence of indication for renal impairment were feasible. In the absence of an indication for renal impairment, LDOM is significantly (*p <* 0.05) associated with the occurrence of pericardial effusion across the different dose ranges (≤10, ≤5, ≤2.5 and ≤1.25 mg). Again, the signal detected for less than three cases should be interpreted with caution for reasons explained earlier.

**TABLE 3:** Reporting odds ratio (ROR) for association between pericardial effusion and LDOM in the presence and absence of an indication for renal and cardiac impairment, across 2016–2023 (inclusive).

Stratum	No. of cases	ROR	Lower bound of 95% CI	Upper bound of 95% CI	No. of cases	ROR	Lower bound of 95% CI	Upper bound of 95% CI	No. of cases	ROR	Lower bound of 95% CI	Upper bound of 95% CI	No. of cases	ROR	Lower bound of 95% CI	Upper bound of 95% CI
	LDOM ≤1.25 mg				LDOM ≤2.5 mg				LDOM ≤5 mg				LDOM ≤10 mg			
Pericardial effusion (in absence of an indication for renal impairment)	1	16.42	2.296	117.456	6	13.308	5.964	29.697	11	18.448	10.19	33.399	19	19.584	12.465	30.769
	*n* = 97 935 508				*n* = 97 936 339				*n* = 97 936 652				*n* = 97 937 452			
	*n* ^c^ = 130				*n* ^c^ = 961				*n* ^c^ = 1274				*n* ^c^ = 2074			
Pericardial effusion (in absence of an indication for cardiac impairment)	1	18.123	2.532	129.742	2	5.336	1.332	21.371	4	8.259	3.094	22.048	10	12.607	6.77	23.475
	*n* = 96 555 811				*n* = 96 556 490				*n* = 96 556 724				*n* = 96 557 385			
	*n* ^c^ = 118				*n* ^c^ = 797				*n* ^c^ = 1031				*n* ^c^ = 1692			
Pericardial effusion (in presence of an indication for cardiac impairment)		*—*	*—*	*—*	4	49.422	18.274	133.664	7	58.631	27.548	124.787	9	47.695	24.521	92.77
	*—*				*n* = 1 386 515 *n* ^c^ = 164				*n* = 1 386 594 *n* ^c^ = 243				*n* = 1 386 733 n^c^ = 382			
	*—*				Test of homogeneity, *p <* 0.05				Test of homogeneity, *p <* 0.05				Test of homogeneity, *p <* 0.05			

*Note:* No. of cases: Number of cases correspond to number of reports retrieved for the respective AE and the respective dose range of minoxidil. The information presented in this table was produced from analyses of the US Food and Drug Administration Adverse Event Reporting System (FAERS) database. This table shows the association between LDOM and pericardial effusion with and without an indication for cardiac impairment. The test for homogeneity corresponds to Breslow‐Day test; this test was conducted to determine whether the stratified RORs are significantly different between the two strata (i.e., presence and absence of an indication for cardiac impairment). Each ROR in this table correspond to a statistically significant (*p <* 0.05) association.

Abbreviations: CI, confidence interval, LDOM, low‐dose oral minoxidil; mg, milligram; *n*, number of observations/sample size ROR computation was based from *n* ^c^, number of reports retrieved for the respective dose range and respective strata (e.g., number of reports retrieved for ≤10 mg across those for whom there was indication for cardiac impairment = 1692).

## 4 Discussion

Our work is the first FAERS‐based pharmacovigilance study for AEs that previous case reports stated were associated with LDOM. In addition to the ≤10 mg dose range, we analyzed the commonly prescribed dose ranges, ≤1.25, ≤2.5, and ≤5 mg.

There are limitations which are inherent to pharmacovigilance databases, including FAERS [8]. A drawback is possible under‐reporting of AEs; reasons include: (i) fear that reporting could have medicolegal repercussions, (ii) belief that some AEs have already been documented by the time the drug is in the market (i.e., passed Phase 3 trials), and (iii) uncertainty of whether the AE is linked to the drug [19]. With LDOM, it cannot be ruled out that the signals for pericardial effusion are a result of (unmeasured) confounding; the FAERS database is presently not structured to document medical information such as coexisting conditions. Thus, our works—as well as related ones—support the update of FAERS to incorporate comorbidity information because it would help analyses and inference making.

Despite the limitations of postmarketing surveillance data, pharmacovigilance databases—as Nguyen et al. put it—are a “cornerstone” for the evaluation of drug safety [8]. While findings from such databases may not be solely used to make causal inferences regarding a drug (i.e., a cause) and an AE (i.e., an effect), postmarketing surveillance data can still be triangulated [20] with results from other study designs to make solid conclusions.

Though Ortega‐Quijano et al.'s study was the first to involve FAERS for LDOM and hair loss, it was solely descriptive and exploratory. Our analytical findings complement those of Ortega‐Quijano et al.'s [9]; we also found a greater preponderance of males across most of the AEs—this could be due to LDOM being used more by men (hence more reporting from men). Unlike Ortega‐Quijano et al.'s (2021) results, our findings serve as evidence for a statistically positive correlation between oral minoxidil and associated AEs from a spontaneously reporting database. Furthermore, we examined a greater variety of oral minoxidil as we did not restrict our analyses by brand names.

A signal was detected for hypertrichosis only with the highest dose range (≤10 mg) (Table 1). The signal for hypertrichosis is dose‐dependent and is consistent with previous reports [21, 22]. Sanabria et al. found that hypertrichosis is significantly more common at younger ages [23]; our study also supports this finding.

We detected a signal for pericardial effusion across the four dose ranges (≤ 1.25 mg, ≤ 2.5, ≤ 5, and ≤10 mg)—and even in the absence of an indication for renal and cardiac impairment. Additionally, we ran a multivariable regression model where the outcome variable corresponded to pericardial effusion (i.e., report of pericardial effusion vs. no report of pericardial effusion) and the explanatory variables were (1) age (in years) and (2) drug (i.e., LDOM up to respective dose range vs. all other drugs). For each dose range (i.e., ≤1.25, ≤2.5, and ≤5 mg), a signal was still detected for pericardial effusion even after adjusting for age (data not shown). In other words: our regression analyses showed that even after accounting for the relationship between age and reporting of pericardial effusion, LDOM (at any of the dose ranges) is associated with this adverse event.

Small pericardial effusions may be asymptomatic. As the pericardial effusion gets larger, it may become symptomatic causing chest tightness, shortness of breath, pleuritic pain (pericarditis), dizziness, tachycardia, chest pain, cardiac tamponade, and peripheral edema. If a pericardial effusion is clinically suspected an echocardiogram or CT/MRI should be considered (chest x‐ray or ECG may not be discriminatory enough).

When there is an indication for cardiac impairment there is a much higher chance for the development of pericardial effusion than in the absence of an indication for cardiac impairment. Subjects with cardiac disease may benefit from (i) evaluation by a cardiologist before being prescribed oral minoxidil and (ii) be monitored closely during therapy.

Results from analyses of postmarketing surveillance data cannot be solely used to make causal inferences. From the FAERS database, in the patients receiving oral minoxidil ≤10 mg/day, there was a report of a positive dechallenge (the AE abated when the drug was stopped) in 8 of 19 reports. In the other 11 cases, there was no information provided on the status postdechallenge. There were no reported cases of positive rechallenge (recurrence of the AE when the drug was restarted). Data from more studies could either verify a causal link or show that the associations from observational data were actually confounded findings [24]. Findings from our analyses warrant further evaluation of the safety of LDOM. Over time, collection of more FAERS data can help in profiling LDOM users especially for those that report pericardial effusion.

The data suggest that some signals are more likely to be seen at higher oral minoxidil dose ranges (≤10 mg) (for example, hypertrichosis); however, other signals (e.g., pericardial effusion) may be idiosyncratic and have been captured at even lower dose ranges (Table 1). Our analysis suggests that other risk factors for the development of pericardial effusion may include the age of the subject and the presence of an indication for cardiac and renal impairment [25]. The number of reports in the literature of pericardial effusion with oral minoxidil at a dose of ≤5 mg is low. It may be prudent for an international registry to be created so that the adverse effects of oral minoxidil can be documented to get a better sense of the frequency and seriousness of cardiac adverse effects. With continued use of oral minoxidil more safety data will accumulate; a reasonable approach would be to restrict the daily dose to ≤5 mg/day when using off‐label (e.g., as in when treating alopecia). Findings from future studies would be needed to solidify our recommendation of ≤5 mg daily.

## 5 Conclusions

This FAERS‐based analytical study detected signals for six potential LDOM‐associated adverse events: pericardial effusion, tachycardia, peripheral edema, hypertrichosis, fluid retention, and headache. Further studies are needed to confirm that LDOM actually causes these adverse events. Until then, it is recommended to use LDOM at the lowest effective dose, ideally not exceeding 5 mg/day.

## Author Contributions

A.K.G.: conceptualization (equal), writing – review and editing (equal). H.A.‐Q.: conceptualization (supporting), writing – original draft (supporting). G.W.: writing – original draft (supporting), writing – review and editing (equal). A.T.: conceptualization (supporting), writing – original draft (supporting). V.P.: conceptualization (supporting), writing – original draft (supporting), writing – review and editing (equal). M.A.B.: conceptualization (supporting), writing – original draft (lead). M.T.: conceptualization (equal), writing – original draft (lead).

## Ethics Statement

Approval from a research ethics board was not required because all our data were already de‐identified.

## Conflicts of Interest

A.K.G., M.T., H.A.Q., G.W., A.T., and M.A.B. have no conflicts of interest to declare. V.P. has received grants from AbbVie, Bausch Health, Celgene, Eli Lilly, Incyte, Janssen, LEO Pharma, L'Oréal, Novartis, Organon, Pfizer, Sandoz, and Sanofi, received payment or honoraria for speaking engagement from Sanofi China, participated on an advisory board for LEO Pharma, Novartis, Sanofi, and Union Therapeutics, and received equipment donation from L'Oréal. V.P. declare that the interests do not affect the objectivity or integrity of this article.

## Supporting information


Data S1.

## Data Availability

Data can be made available upon request.

## References

[1] A. N. Sharma , L. Michelle , M. Juhasz , et al., “Low‐Dose Oral Minoxidil as Treatment for Non‐Scarring Alopecia: A Systematic Review,” International Journal of Dermatology 59, no. 8 (2020): 1013–1019. 32516434 10.1111/ijd.14933

[2] S. Vano‐Galvan , R. Pirmez , A. Hermosa‐Gelbard , et al., “Safety of Low‐Dose Oral Minoxidil for Hair Loss: A Multicenter Study of 1404 Patients,” Journal of the American Academy of Dermatology 84, no. 6 (2021): 1644–1651. 33639244 10.1016/j.jaad.2021.02.054

[3] M. A. Alpert and J. H. Bauer , “Rapid Control of Severe Hypertension With Minoxidil,” Archives of Internal Medicine 142, no. 12 (1982): 2099–2104. 6753777

[4] A. Villani , G. Fabbrocini , J. Ocampo‐Candiani , et al., “Review of Oral Minoxidil as Treatment of Hair Disorders: In Search of the Perfect Dose,” Journal of the European Academy of Dermatology and Venereology 3 (2021): 1485–1492. 10.1111/jdv.17216 33660357

[5] A. K. Gupta , M. Talukder , M. Venkataraman , et al., “Minoxidil: A Comprehensive Review,” Journal of Dermatological Treatment 33, no. 4 (2022): 1896–1906. 34159872 10.1080/09546634.2021.1945527

[6] R. A. Beach , “Case Series of Oral Minoxidil for Androgenetic and Traction Alopecia: Tolerability & the Five C's of Oral Therapy,” Dermatologic Therapy 31, no. 6 (2018): e12707. 30246901 10.1111/dth.12707 PMC6586015

[7] P. M. Coloma , G. Trifirò , V. Patadia , et al., “Postmarketing Safety Surveillance: Where Does Signal Detection Using Electronic Healthcare Records fit Into the big Picture?,” Drug Safety 36, no. 3 (2013): 183–197. 23377696 10.1007/s40264-013-0018-x

[8] D. D. Nguyen , M. Marchese , E. B. Cone , et al., “Investigation of Suicidality and Psychological Adverse Events in Patients Treated With Finasteride,” JAMA Dermatology 157, no. 1 (2021): 35–42. 33175100 10.1001/jamadermatol.2020.3385 PMC7658800

[9] D. Ortega‐Quijano , J. Jimenez‐Cauhe , D. Fernandez‐Nieto , et al., “Comment on “Low Dose Oral Minoxidil for Treating Alopecia: A 3‐Year North American Retrospective Case Series”: Adding Further Evidence About Side Effects,” Journal of the American Academy of Dermatology 84, no. 5 (2021): e237–e238. 33359596 10.1016/j.jaad.2020.12.041

[10] E. Von Elm , D. G. Altman , M. Egger , et al., “The Strengthening the Reporting of Observational Studies in Epidemiology (STROBE) Statement: Guidelines for Reporting Observational Studies,” Lancet 370, no. 9596 (2007): 1453–1457. 18064739 10.1016/S0140-6736(07)61602-X

[11] M. Fusaroli , F. Salvo , B. Begaud , et al., “The Reporting of a Disproportionality Analysis for Drug Safety Signal Detection Using Individual Case Safety Reports in PharmacoVigilance (READUS‐PV): Development and Statement,” Drug Safety 47, no. 6 (2024): 575–584. 38713346 10.1007/s40264-024-01421-9 PMC11116242

[12] Y. Wang , B. Zhao , H. Yang , and Z. Wan , “A Real‐World Pharmacovigilance Study of FDA Adverse Event Reporting System Events for Sildenafil,” Andrology 12, no. 4 (2024): 785–792. 37724699 10.1111/andr.13533

[13] E. G. Brown , L. Wood , and S. Wood , “The Medical Dictionary for Regulatory Activities (MedDRA),” Drug Safety 20, no. 2 (1999): 109–117. 10082069 10.2165/00002018-199920020-00002

[14] H. Wickham , R. François , L. Henry , et al., “dplyr: A Grammar of Data Manipulation” 2023, https://dplyr.tidyverse.org.

[15] G. Grothendieck , “sqldf: Manipulate R Data Frames Using SQL” 2017.

[16] S. Vañó‐Galván , L. Trindade de Carvalho , D. Saceda‐Corralo , et al., “Oral Minoxidil Improves Background Hair Thickness in Lichen Planopilaris,” Journal of the American Academy of Dermatology 84, no. 6 (2021): 1684–1686. 32289397 10.1016/j.jaad.2020.04.026

[17] K. J. Rothman , S. Lanes , and S. T. Sacks , “The Reporting Odds Ratio and Its Advantages Over the Proportional Reporting Ratio,” Pharmacoepidemiology and Drug Safety 13, no. 8 (2004): 519–523. 15317031 10.1002/pds.1001

[18] E. P. van Puijenbroek , A. Bate , H. G. Leufkens , et al., “A Comparison of Measures of Disproportionality for Signal Detection in Spontaneous Reporting Systems for Adverse Drug Reactions,” Pharmacoepidemiology and Drug Safety 11, no. 1 (2002): 3–10. 11998548 10.1002/pds.668

[19] L. Hazell and S. A. Shakir , “Under‐Reporting of Adverse Drug Reactions: A Systematic Review,” Drug Safety 29, no. 5 (2006): 385–396. 16689555 10.2165/00002018-200629050-00003

[20] D. A. Lawlor , K. Tilling , and S. G. Davey , “Triangulation in Aetiological Epidemiology,” International Journal of Epidemiology 45, no. 6 (2016): 1866–1886. 28108528 10.1093/ije/dyw314 PMC5841843

[21] A. K. Gupta , M. Talukder , A. Shemer , et al., “Low‐Dose Oral Minoxidil for Alopecia: A Comprehensive Review,” Skin Appendage Disorders 9, no. 6 (2023): 423–437. 38376087 10.1159/000531890 PMC10806356

[22] A. K. Gupta , D. Hall , M. Talukder , et al., “There is a Positive Dose‐Dependent Association Between Low‐Dose Oral Minoxidil and Its Efficacy for Androgenetic Alopecia: Findings From a Systematic Review With Meta‐Regression Analyses,” Skin Appendage Disorders 8, no. 5 (2022): 355–361. 36161084 10.1159/000525137 PMC9485924

[23] B. Sanabria , V. T. de Nardo , H. A. Miot , and P. M. Ramos , “Adverse Effects of Low‐Dose Oral Minoxidil for Androgenetic Alopecia in 435 Patients,” Journal of the American Academy of Dermatology 84, no. 4 (2021): 1175–1178. 33253848 10.1016/j.jaad.2020.11.035

[24] T. J. VanderWeele , “Explanation in Causal Inference: Developments in Mediation and Interaction,” International Journal of Epidemiology 45, no. 6 (2016): 1904–1908. 27864406 10.1093/ije/dyw277 PMC6373498

[25] M. J. Krantz and J. B. Byrd , “Pericardial Effusion in Renal Disease: To Tap or Not to Tap,” Cardiology 120, no. 4 (2011): 204–208. 22286154 10.1159/000335482

