# Supplementary material for: Low‐Dose Oral Minoxidil and Associated Adverse Events: Analyses of the FDA Adverse Event Reporting System (FAERS) With a Focus on Pericardial Effusions
Source: J Cosmet Dermatol. 2024 Sep 26;24(1):e16574. doi: 10.1111/jocd.16574 (PMC11743066; doi:10.1111/jocd.16574)
Supplement: Supplementary file 1 — Data S1. [file JOCD-24-e16574-s001.docx]

# Appendix A: Characteristics of reports associated with LDOM ≤ 10 mg and the 10 AEs across 2016 to 2023 (inclusive)

| **Dizziness** | |
| --- | --- |
| **Sex (%)** |  |
| Female | 47.4% |
| Male | 52.6% |
| **Age (years)** |  |
| Mean (SD) | 65.211 (±8.158) |
| **Profession of reporter** |  |
| Consumer | 45% |
| Other health-professional | 23% |
| Physician | 23% |
| Pharmacist | 9% |
| **Fluid retention** | |
| **Sex (%)** |  |
| Female | 28.6% |
| Male | 71.4% |
| **Age (years)** |  |
| Mean (SD) | 52.764 (±21.827) |
| **Profession of reporter** |  |
| Consumer | 57% |
| Pharmacist | 29% |
| Other health-professional | 14% |
| **Headache** | |
| **Sex (%)** |  |
| Female | 52.0% |
| Male | 48.0% |
| **Age (years)** |  |
| Mean (SD) | 49.619 (±16.657) |
| **Profession of reporter** |  |
| Other health-professional | 56% |
| Consumer | 22% |
| Physician | 19% |
| Pharmacist | 4% |
| **Hypertrichosis** | |
| **Sex (%)** |  |
| Female | 33.3% |
| Male | 66.7% |
| **Age (years)** |  |
| Mean (SD) | 28.667 (±39.260) |
| **Profession of reporter** |  |
| Pharmacist | 67% |
| Consumer | 33% |
| **Hypotension** | |
| **Sex (%)** |  |
| Female | 38.5% |
| Male | 61.5% |
| **Age (years)** |  |
| Mean (SD) | 52.111 (±23.008) |
| **Profession of reporter** |  |
| Physician | 31% |
| Consumer | 23% |
| Other health-professional | 23% |
| Pharmacist | 23% |
| **Insomnia** | |
| **Sex (%)** |  |
| Female | 50.0% |
| Male | 50.0% |
| **Age (years)** |  |
| Mean (SD) | 62.750 (±9.979) |
| **Profession of reporter** |  |
| Consumer | 71% |
| Other health-professional | 14% |
| Physician | 14% |
| **Pericardial effusion** | |
| **Sex (%)** |  |
| Female | 16.7% |
| Male | 83.3% |
| **Age (years)** |  |
| Mean (SD) | 58.647 (±17.113) |
| **Profession of reporter** |  |
| Other health-professional | 32% |
| Pharmacist | 32% |
| Physician | 26% |
| Consumer | 5% |
| - | 5% |
| **Peripheral edema** | |
| **Sex (%)** |  |
| Female | 8.3% |
| Male | 91.7% |
| **Age (years)** |  |
| Mean (SD) | 65.417 (±10.808) |
| **Profession of reporter** |  |
| Pharmacist | 27% |
| Consumer | 20% |
| Other health-professional | 20% |
| Physician | 20% |
| Lawyer | 7% |
| - | 7% |
| **Tachycardia** | |
| **Sex (%)** |  |
| Female | 88.9% |
| Male | 11.1% |
| **Age (years)** |  |
| Mean (SD) | 50.000 (±21.576) |
| **Profession of reporter** |  |
| Other health-professional | 58% |
| Pharmacist | 21% |
| Physician | 16% |
| Consumer | 5% |

**Abbreviations:**

LDOM = low-dose oral minoxidil, SD = standard deviation

**Notes:**

This table summarizes the distribution of age and sex across reports of LDOM (≤ 10 mg) for each adverse event; information regarding reporters’ profession is also provided. Data were not available for periorbital edema.

# Appendix B: Overview of data mining for case retrieval

## Section 1: Overview for ‘creation of ‘main dataset’


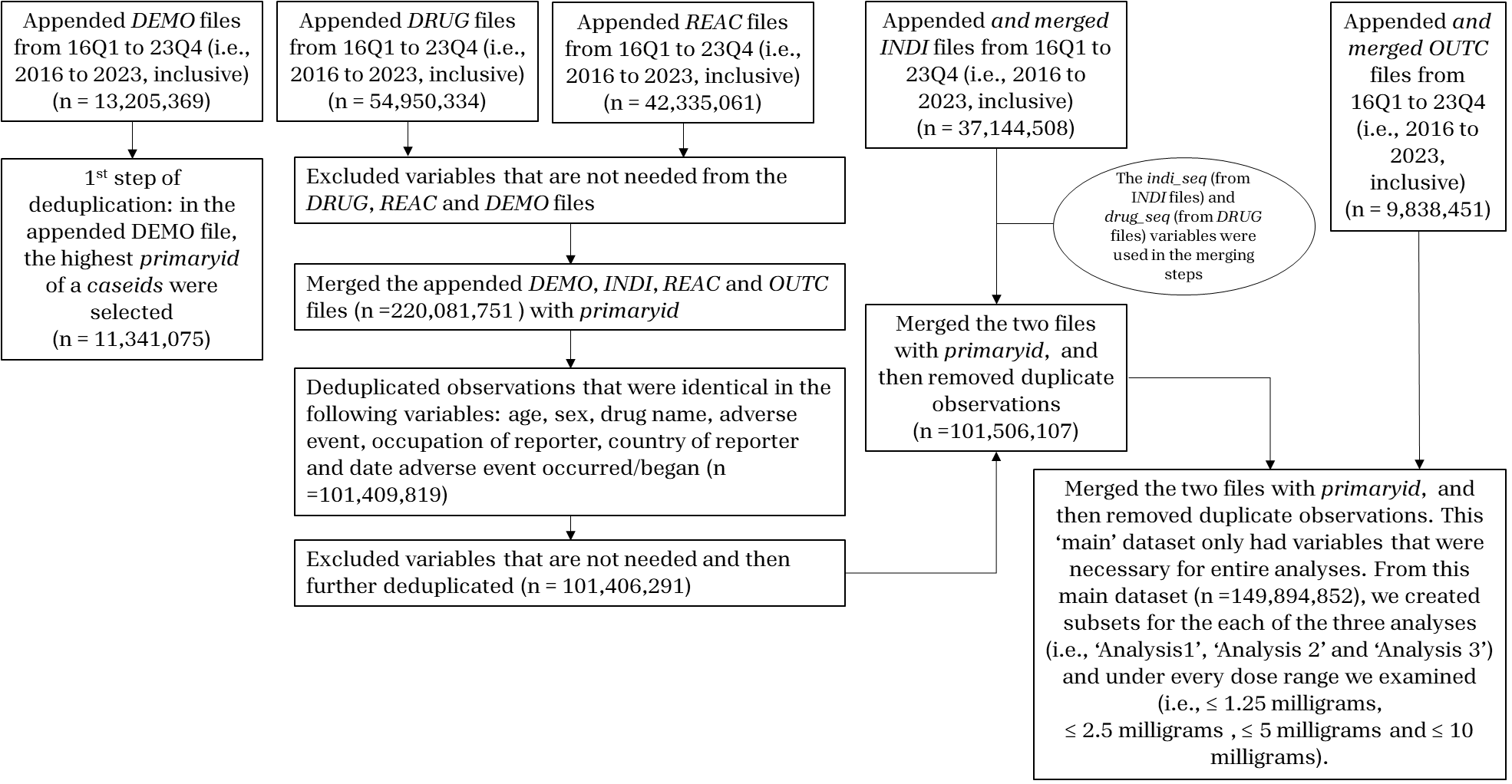


## Section 2: Overview for creation of ‘subsets’ of main dataset—according to respective analyses

###
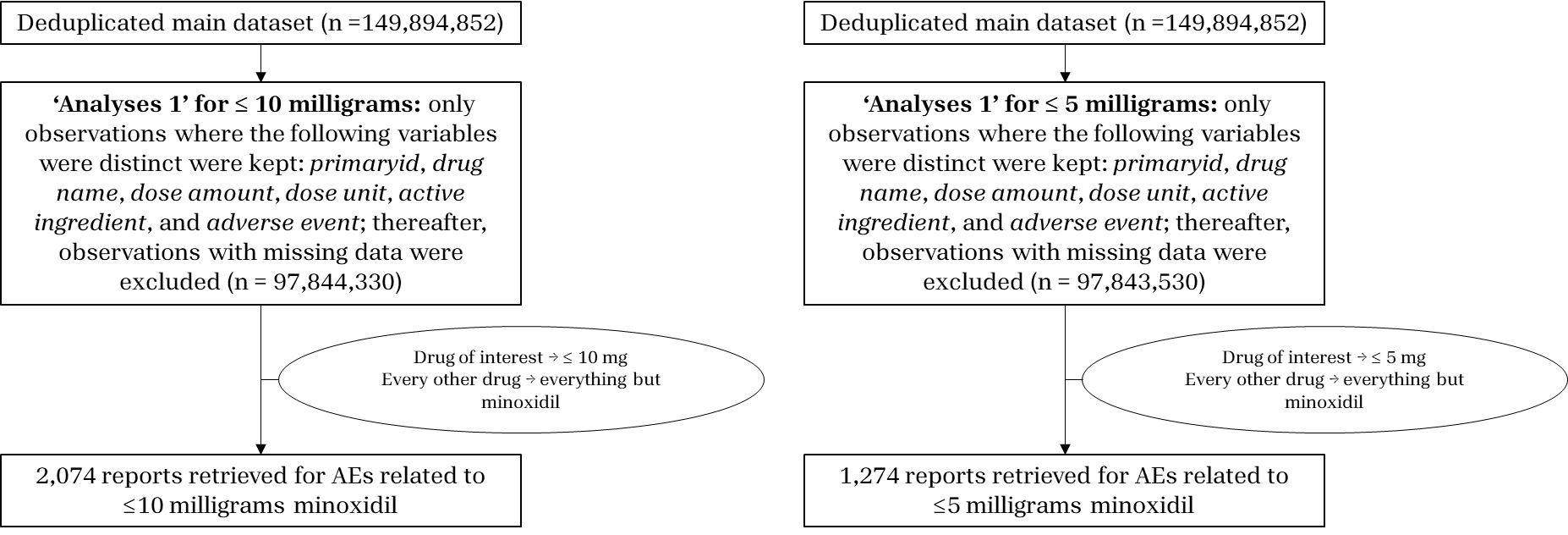
‘Analyses 1’


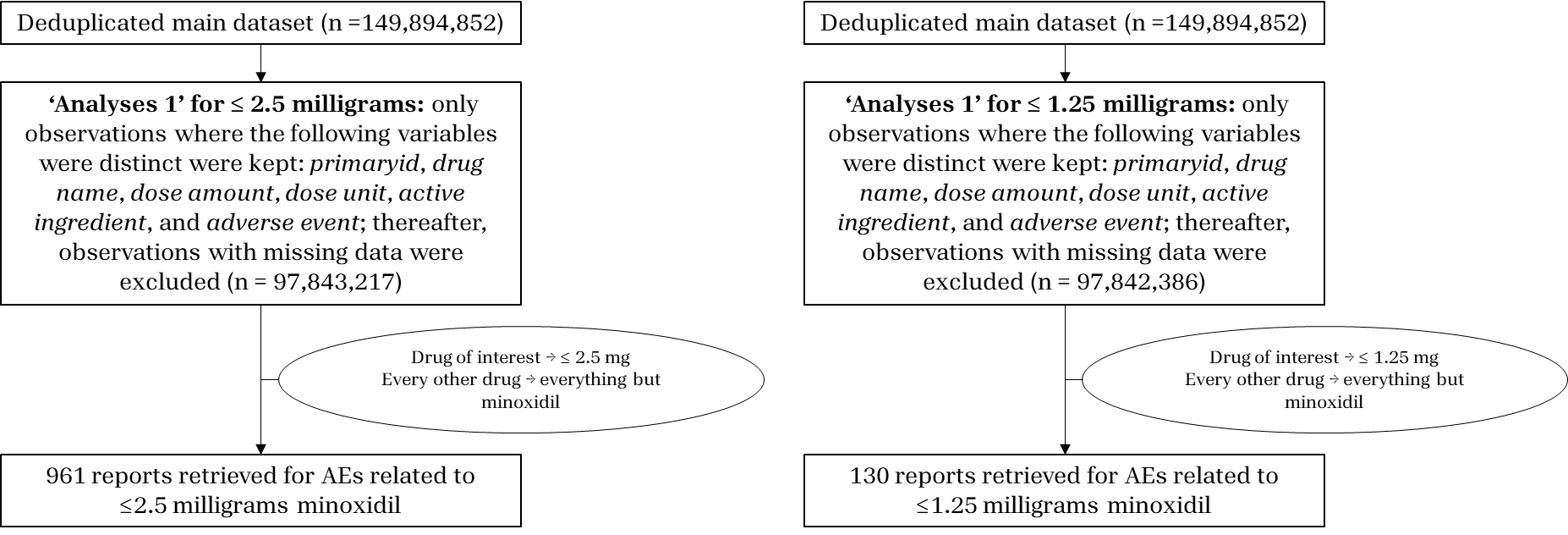


### ‘Analyses 2’


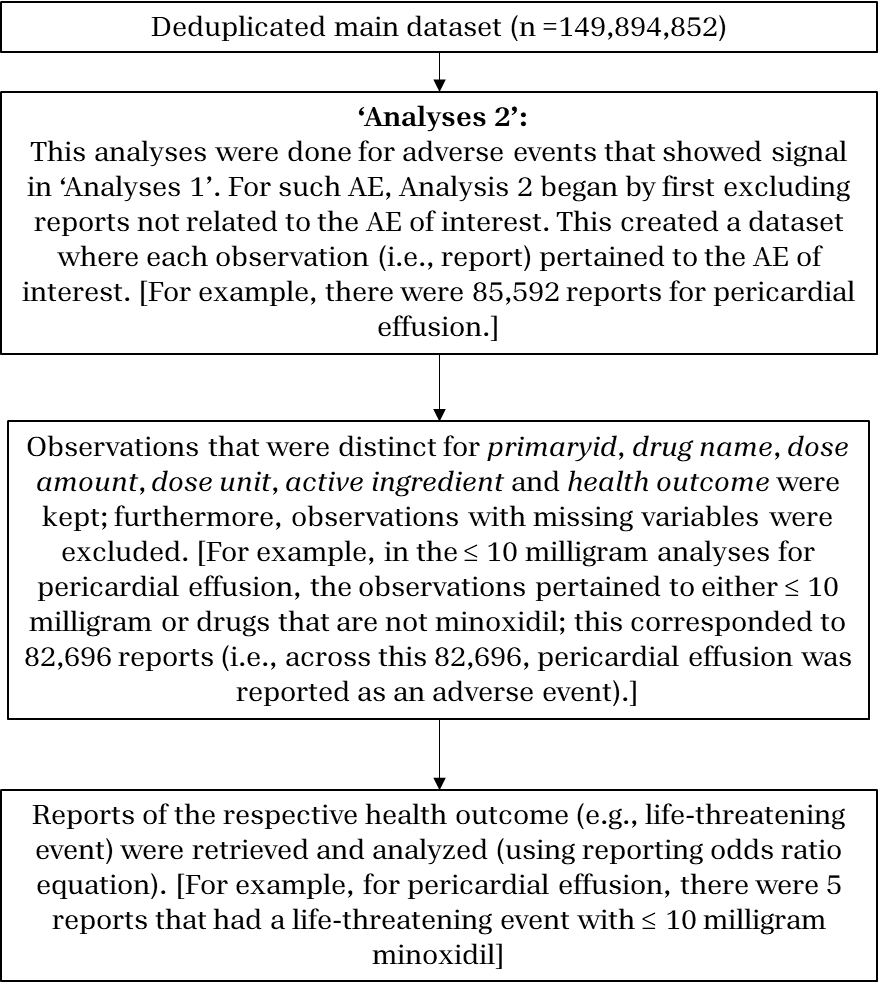


### Analyses 3


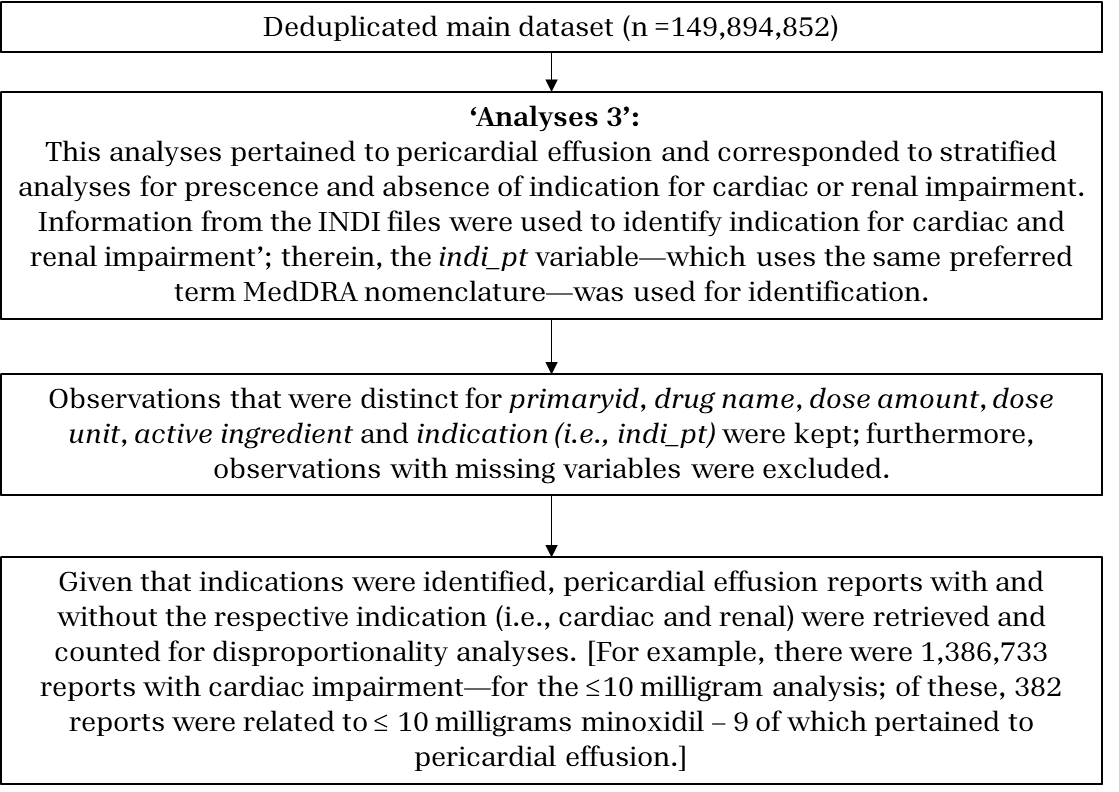


**eTable 1.** Occurrence of adverse events from using and not using LDOM ≤1.5 mg, across 2016 – 2023 (inclusive) (n = 97,842,386)

| AE | LDOM use | Non-use of LDOM |
| --- | --- | --- |
| Dizziness vs. all other AEs | - | - |
| Fluid retention vs. all other AEs | - | - |
| Headache vs. all other AEs | 7.44% | 0.89% |
| Hypertrichosis vs. all other AEs | - | - |
| Hypotension vs. all other AEs | 1.57% | 0.42% |
| Insomnia vs. all other AEs | - | - |
| Pericardial effusion vs. all other AEs | 0.78% | 0.05% |
| Periorbital edema vs. all other AEs | - | - |
| Peripheral edema vs. all other AEs | 0.76% | 0.21% |
| Tachycardia vs. all other AEs | 7.44% | 0.25% |

**Abbreviations:**

AE = adverse event,

mg = milligram,

n = number of observations/sample size,

LDOM = low-dose oral minoxidil

**Notes:**

- The information in this table was based on the United States Food and Drug Administration Adverse Event Reporting System (FAERS) database. Herein, we presented a descriptive summary for the relative occurrence of 10 respective AEs associated with use of LDOM.
- To understand the content of this table, below is an explanation for the third row:
- Across reports for LDOM ≤ 1.5 mg:

the occurrence of Headache ÷ the occurrence of all other AEs (i.e., everything but Headache) = 7.44%

- Across reports for all other drugs:

the occurrence of Headache ÷ the occurrence of all other AEs (i.e., everything but Headache) = 0.89%

**eTable 2.** Occurrence of adverse events from using and not using LDOM ≤2.5 mg, across 2016 – 2023 (inclusive)(n = 97,843,217)

| AE | LDOM use | Non-use of LDOM |
| --- | --- | --- |
| Dizziness vs. all other AEs | 1.06% | 0.74% |
| Fluid retention vs. all other AEs | 0.21% | 0.11% |
| Headache vs. all other AEs | 1.27% | 0.89% |
| Hypertrichosis vs. all other AEs | - | - |
| Hypotension vs. all other AEs | 0.42% | 0.42% |
| Insomnia vs. all other AEs | 0.53% | 0.47% |
| Pericardial effusion vs. all other AEs | 0.63% | 0.05% |
| Periorbital edema vs. all other AEs | - | - |
| Peripheral edema vs. all other AEs | 0.84% | 0.21% |
| Tachycardia vs. all other AEs | 1.38% | 0.25% |

**Abbreviations:**

AE = adverse event,

mg = milligram,

n = number of observations/sample size,

LDOM = low-dose oral minoxidil

**Notes:**

- The information in this table was based on the United States Food and Drug Administration Adverse Event Reporting System (FAERS) database. Herein, we presented a descriptive summary for the relative occurrence of 10 respective AEs associated with use of LDOM.
- To understand the content of this table, below is an explanation for the first row:
- Across reports for LDOM ≤ 2.5 mg:

the occurrence of Dizziness ÷ the occurrence of all other AEs (i.e., everything but Dizziness) = 1.06%

- Across reports for all other drugs:

the occurrence of Dizziness ÷ the occurrence of all other AEs (i.e., everything but Dizziness) = 0.74%

**eTable 3.** Occurrence of adverse events from using and not using LDOM ≤ 5 mg, across 2016 – 2023 (inclusive) (n =97,843,530)

| AE | LDOM use | Non-use of LDOM |
| --- | --- | --- |
| Dizziness vs. all other AEs | 1.04% | 0.74% |
| Fluid retention vs. all other AEs | 0.40% | 0.11% |
| Headache vs. all other AEs | 1.20% | 0.89% |
| Hypertrichosis vs. all other AEs | - | - |
| Hypotension vs. all other AEs | 0.40% | 0.42% |
| Insomnia vs. all other AEs | 0.40% | 0.47% |
| Pericardial effusion vs. all other AEs | 0.88% | 0.05% |
| Periorbital edema vs. all other AEs | - | - |
| Peripheral edema vs. all other AEs | 0.95% | 0.21% |
| Tachycardia vs. all other AEs | 1.20% | 0.25% |

**Abbreviations:**

AE = adverse event, mg = milligram, n = number of observations/sample size, LDOM = low-dose oral minoxidil

**Notes:**

- The information in this table was based on the United States Food and Drug Administration Adverse Event Reporting System (FAERS) database. Herein, we presented a descriptive summary for the relative occurrence of 10 respective AEs associated with use of LDOM.
- To understand the content of this table, below is an explanation for the first row:
- Across reports for LDOM ≤ 5 mg:

the occurrence of Dizziness ÷ the occurrence of all other AEs (i.e., everything but Dizziness) = 1.04%

- Across reports for all other drugs:

the occurrence of Dizziness ÷ the occurrence of all other AEs (i.e., everything but Dizziness) = 0.74%

**eTable 4.** Occurrence of adverse events from using and not using LDOM ≤ 10 mg, across 2016 – 2023 (inclusive) (n =97,844,330)

| AE | LDOM use | Non-use of LDOM |
| --- | --- | --- |
| Dizziness vs. all other AEs | 1.08% | 0.74% |
| Fluid retention vs. all other AEs | 0.34% | 0.11% |
| Headache vs. all other AEs | 1.34% | 0.89% |
| Hypertrichosis vs. all other AEs | 0.15% | 0.01% |
| Hypotension vs. all other AEs | 0.64% | 0.42% |
| Insomnia vs. all other AEs | 0.34% | 0.47% |
| Pericardial effusion vs. all other AEs | 0.93% | 0.05% |
| Periorbital edema vs. all other AEs | - | - |
| Peripheral edema vs. all other AEs | 0.73% | 0.21% |
| Tachycardia vs. all other AEs | 0.93% | 0.25% |

**Abbreviations:**

AE = adverse event, mg = milligram, n = number of observations/sample size, LDOM = low-dose oral minoxidil

**Notes:**

- The information in this table was based on the United States Food and Drug Administration Adverse Event Reporting System (FAERS) database. Herein, we presented a descriptive summary for the relative occurrence of 10 respective AEs associated with use of LDOM.
- To understand the content of this table, below is an explanation for the first row:
- Across reports for LDOM ≤ 10 mg:

the occurrence of Dizziness ÷ the occurrence of all other AEs (i.e., everything but Dizziness) = 1.08%

- Across reports for all other drugs:

the occurrence of Dizziness ÷ the occurrence of all other AEs (i.e., everything but Dizziness) = 0.74%
